# N6-methyladenosine methylation regulators can serve as potential biomarkers for endometriosis related infertility

**DOI:** 10.17305/bb.2024.11311

**Published:** 2024-12-25

**Authors:** Yalun He, Jie Ding, Tonglin Bai, Yangshuo Li, Xiaolan Liang, Yiming Chen, Yi Lin, Wen Cheng, Chaoqin Yu

**Affiliations:** 1 Department of Gynecology of Traditional Chinese Medicine, The First Affiliated Hospital of Naval Medical University, Shanghai, China; 2 Department of Physiotherapy of Traditional Chinese Medicine, Beidaihe Rehabilitation and Recuperation Center, The People’s Liberation Army Joint Logistic Support Force, Qinhuangdao, China; 3 Department of Reproductive Medicine, Shuguang Hospital affiliated to Shanghai University of Traditional Chinese Medicine, Shanghai, China; 4 Shanghai University of Traditional Chinese Medicine, Shanghai, China

**Keywords:** N6-methyladenosine, m6A, endometriosis, EMS, immune system, infertility

## Abstract

Endometriosis (EMS) is a chronic inflammatory disease frequently associated with infertility. N6-methyladenosine (m6A) methylation, the most common form of methylation in eukaryotic mRNAs, has gained attention in the study of female reproductive diseases, including EMS and infertility. This study aimed to investigate the role of m6A regulators in EMS-related infertility. To begin, specific m6A regulators were identified by analyzing the GSE120103 dataset, followed by receiver operating characteristic (ROC) curve analysis. A nomogram model was then constructed, and unsupervised clustering of m6A regulators was performed to identify distinct m6A molecular clusters. Functional enrichment analysis of differentially expressed genes (DEGs) between these clusters, along with immune cell infiltration analysis, was subsequently conducted. In addition, the single-cell dataset GSE214411 was analyzed to explore the role of m6A regulators in various cell types. Finally, clinical samples were collected, and immunohistochemistry analysis was performed. The study identified seven key m6A regulators with significant diagnostic value for EMS-related infertility and two distinct m6A molecular clusters. Gene Ontology (GO) and Kyoto Encyclopedia of Genes and Genomes (KEGG) analyses of DEGs between the clusters revealed that m6A clustering was strongly associated with immune pathways. Immune cell infiltration analysis further demonstrated that the expression levels of m6A regulators had a notable impact on immune cell infiltration. Single-cell analysis revealed that *HNRNPA2B1* and *HNRNPC* were significantly elevated in endometrial immune cells from infertile EMS patients but notably decreased in stromal cells. Immunohistochemical staining confirmed that HNRNPA2B1 and HNRNPC expression levels were significantly higher in the eutopic endometrium of fertile women compared to ovarian EMS patients. These findings suggest that m6A regulators play critical roles in the development and progression of EMS-related infertility. Notably, *HNRNPA2B1* and *HNRNPC* may serve as potential biomarkers for this condition.

## Introduction

Endometriosis (EMS) is a chronic inflammatory disease characterized by the presence of endometrial tissue outside the uterus. Due to its prolonged course and the primary focus of treatment being symptom relief rather than cure, EMS is regarded as a significant public health issue. It severely impacts women’s quality of life and poses a substantial economic burden [1]. EMS manifests primarily in three forms: peritoneal, ovarian, and deep infiltrating, with the ovarian type accounting for approximately 70% of cases [2]. Patients with EMS often experience symptoms, such as chronic pelvic pain, dysmenorrhea, and infertility, all of which significantly affect their daily lives. Among these symptoms, infertility is particularly prominent. Nearly 10%–15% of women of reproductive age suffer from EMS, and approximately one-third of them experience infertility—double the rate of women without the condition. Additionally, up to 50% of infertile women are diagnosed with EMS [3]. The clinical manifestations of EMS are often misinterpreted as common menstrual symptoms in women of reproductive age. Coupled with the lack of effective non-invasive diagnostic tools, this misinterpretation leads to a delayed diagnosis of EMS—typically by 8–10 years [4]. These challenges underscore the difficulty in diagnosing and treating EMS and its associated infertility. The exact pathogenesis of EMS remains unclear. While the classical theory of menstrual reflux is the most widely recognized, it fails to fully explain the diverse range of EMS manifestations [5]. Other factors, such as epigenetic defects, epithelial cell mutations, inflammation, oxidative stress, and RNA methylation, are also believed to contribute to the progression of EMS [6]. The mechanisms underlying EMS-related infertility are similarly unclear and are thought to result from complex multifactorial interactions [3]. Recent research has suggested that changes in RNA methylation processes may play a key role in EMS-related infertility. RNA methylation, particularly N6-methyladenosine (m6A) methylation, has emerged as a potential mechanism of interest. m6A methylation, the most common form of methylation in eukaryotic mRNAs, regulates various stages of the RNA life cycle, including transcription, maturation, translation, splicing, degradation, and stability [7]. Advances in sequencing technologies have significantly expanded our understanding of m6A methylation, shedding light on its regulatory mechanisms. The m6A regulatory process requires specific m6A regulators to mediate its functions, which are critical to various biological processes. Recent studies have revealed that these m6A regulators also play roles in the development and progression of EMS. For instance, the m6A writer *METTL3* has been found to promote M2 macrophage polarization by activating its target gene *Trib1*. *METTL3* also inhibits the maturation of pri-miR6 in an m6A-dependent manner, enhancing cell migration and invasion, and thereby facilitating EMS progression [8, 9]. Additionally, the loss of *METTL3* has been linked to oocyte maturation failure and impaired fertility, potentially due to the downregulation of m6A methylation levels and the suppression of critical genes involved in steroid hormone synthesis and gonadotropin signaling pathways [10]. However, research on the roles of other m6A regulators in EMS and EMS-related infertility remains limited, and the molecular mechanisms are not yet fully understood. This study analyzed the expression levels of m6A regulators in eutopic endometrium using the GSE120103 dataset from the GSE database. The results identified m6A regulators of significant diagnostic importance for EMS-related infertility and facilitated the construction of a nomogram model based on these regulators. Furthermore, two distinct m6A molecular clusters were identified. Gene Ontology (GO) and Kyoto Encyclopedia of Genes and Genomes (KEGG) analyses of the differentially expressed genes (DEGs) between these two molecular clusters were conducted to provide additional insights. The study also examined the correlation between key candidate m6A regulators and immune cell infiltration by performing immune cell infiltration analysis. Additionally, the GSE214411 single-cell dataset was utilized to analyze the expression levels of these key m6A regulators across various cell populations. Immunohistochemical staining was further performed on eutopic endometrium samples collected from ovarian EMS infertile patients and normal fertile women.

In summary, the findings suggest that *HNRNPA2B1* and *HNRNPC* could serve as potential biomarkers for EMS-related infertility. These biomarkers may improve the ability to predict EMS-related infertility and facilitate timely treatment interventions.

## Materials and methods

### Public database analysis

#### Identification and clinical relevance analysis of key m6A regulators

The dataset GSE120103 was selected as the primary research dataset from the GEO database. It included samples from nine normal fertile women and 18 stage IV ovarian EMS patients (nine fertile and nine infertile). RNA sequencing analysis was performed on endometrial samples from these groups, resulting in the identification of 26 significant m6A regulators [11]. These regulators include 15 readers, such as *ELAVL1* and *FMR1*; nine writers, such as *METTL14* and *METTL16*; and two erasers, *ALKBH5* and *FTO*.

The “limma” package was used for pairwise analysis to identify differential m6A regulators between groups, and Venn diagrams were generated to pinpoint key regulators potentially linked to EMS-related infertility. The “pROC” package was applied to analyze the area under the curve (AUC) values of these key regulators, with an AUC value ≥ 0.8 indicating excellent predictive performance [12]. Based on the identified m6A regulators, a nomogram model was constructed using the “rms” and “rmda” packages in R. A nomogram is a statistical tool used to visualize prediction model outcomes, helping physicians and patients estimate an individual’s prognosis probability based on specific risk factors [13]. Below is a summary of how to interpret the nomogram and its results [13]. Identify variables: The nomogram consists of multiple vertical segments, each representing a predictor variable. Each segment has a corresponding scale, indicating the numerical value or classification of the variable. Determine individual scores: For each predictor variable, find the corresponding score on its segment based on the individual’s actual data. Add up the scores of all variables to calculate the total score. Assess prognostic probability: Using the total score, locate the corresponding position on the “Total Points” segment. Then move vertically down to the “Prognostic Probability” segment to determine the individual’s prognosis probability. To evaluate the model’s accuracy, calibration curves and decision curve analysis (DCA) were employed to assess whether the gene-based model offers clinical decision making and diagnostic benefits for EMS patients.

### Identification of clusters based on key m6A regulators and identification of DEGs between clusters

Based on the candidate key m6A regulators, the “ConsensusClusterPlus” package in R was used to identify m6A clusters in the dataset using maxK (consensus clustering coefficient) ranging from 2 to 9. Principal component analysis (PCA) was used to distinguish m6A clusters. After determining the clusters, the DEGs between the two clusters were screened via the “limma” package in R, with a significance threshold of *P* value < 0.05 and ∣logFC∣ ≥ 2.

### GO and KEGG enrichment analysis of DEGs

The functional enrichment analysis of the DEGs identified in Section 2.1.2 was conducted using GO and KEGG analyses via the “clusterProfiler,” “org.Hs.eg.db,” “enrichmentplot,” and “ggplot” packages in R. These analyses unveiled potential molecular mechanisms associated with the DEGs across different m6A clusters in EMS.

### Immune cell infiltration analysis

To identify the abundance of various immune cells in the eutopic endometrium across different m6A clusters in EMS, this study employed single-sample gene set enrichment analysis (ssGSEA) to analyze gene expression profiles and assess correlations between immune cells and genes. To visualize the levels of immune cell infiltration in the different clusters and their associations with the expression of key m6A regulators, the study made use of the R packages “reshape2,” “ggplot2,” “limma,” “GSEABase,” and “GSVA.”

### Single-cell analysis

To further validate the results of bulk RNA sequencing, this study incorporated a single-cell dataset (GSE214411) that included eutopic endometrium samples from six stage I or II EMS infertile patients and seven normal fertile women [14]. First, the raw data were processed using the “mkfastq” application of Cell Ranger. Data quality control was then conducted using the Seurat package with the following filtering criteria [14]: (1) genes expressed in fewer than three cells were excluded; (2) only cells expressing at least 200 genes were retained; (3) cells with more than 20% mitochondrial gene content were removed to eliminate broken cells; and (4) erythrocyte and cell cycle-related genes were excluded. To address batch effects, the Harmony clustering method was applied, while dimensionality reduction and visualization were performed using the UMAP algorithm, which displayed cell clusters in two dimensions. Cluster-specific marker genes were identified based on enriched genes within each cluster, referencing markers from the relevant literature. Additionally, the violin and sierra figure functions in Seurat were used to plot and compare differences in seven key m6A regulators between the normal fertile group and the EMS infertile group, as well as variations across cell populations.

### Clinical sample analysis

#### Inclusion and exclusion criteria for clinical sample collection

The eutopic endometrium samples used in this study were collected from the Reproductive Center of the First Affiliated Hospital of Naval Medical University between July 1, 2023, and November 30, 2023. Samples were taken from two groups: stage III or IV infertile ovarian EMS patients undergoing assisted reproductive technology (three patients) and normal control women undergoing assisted reproductive technology due to male infertility (three patients). The study received approval from the Ethics Committee of the First Affiliated Hospital of Naval Medical University (CHEC2019-100) and was conducted in compliance with the Declaration of Helsinki. The inclusion criteria for infertile EMS patients were as follows: patients diagnosed with ovarian EMS through pathology and meeting the following conditions: (1) aged between 25 and 35 years; (2) cohabitating for one year or more, engaging in regular sexual activity without using contraception, and without achieving pregnancy; and (3) male partner with normal semen analysis according to KRUGER standards. For female controls undergoing assisted reproductive technology due to male infertility, the inclusion criteria were as follows: (1) healthy women aged between 25 and 35 years; (2) male partner diagnosed with infertility based on the “WHO Manual for the Standardized Investigation and Diagnosis of the Infertile Couple” by the World Health Organization; and (3) cohabitating for one year or more, engaging in regular sexual activity without using contraception, and unable to conceive due to male infertility. The study’s exclusion criteria included: Ovulatory disorders: Conditions, such as polycystic ovary syndrome (PCOS), adenomyosis, hyperthyroidism, systemic lupus erythematosus (SLE), hyperprolactinemia, or other autoimmune diseases. Infections: Presence of human immunodeficiency virus (HIV) infection or any other active infection. Tubal Infertility: Infertility caused by tubal factors. Medical History: A history of tuberculosis, pelvic surgery, radiotherapy, or chemotherapy. Smoking: Individuals with a smoking habit. Medication Use: Use of hormonal medications or hormone/nonhormone anti-inflammatory drugs within three months before the consultation. Contraindications: Any contraindications to ovarian stimulation treatments.

### Immunohistochemistry of eutopic endometrium

Endometrial tissue was first fixed in 10% formalin, embedded in paraffin, and sectioned. Following dewaxing and dehydration, the sections were incubated in 3% hydrogen peroxide for 30 min to block endogenous nonspecific peroxidase activity. Immunohistochemistry was performed following a standard protocol. Samples were incubated overnight at 4 ^∘^C with hnRNP C1/C2 rabbit polyclonal antibody (Biodragon, Catalogue No. BD-PT2195) and hnRNP A2/B1 rabbit polyclonal antibody (Biodragon, Catalogue No. BD-PT2193), followed by PBS washing. Next, the sections were incubated with HRP-conjugated anti-rabbit/mouse secondary antibody (Shanghai Wellbio Technology Co., China, #WB0177/#WB0176) at 37 ^∘^C for 45 min. Subsequently, all slides were treated with 3,3’-diaminobenzidine tetrahydrochloride (Maxim, China) for 6 min and counterstained with haematoxylin. After dehydration in absolute ethanol and mounting with neutral resin, the samples were observed and imaged under a Leica microscope (Germany).

### Ethical statement

This study was approved by the Ethics Committee of the First Affiliated Hospital of Naval Medical University (CHEC2019-100) and was conducted in accordance with the Declaration of Helsinki.

## Results

### Expression features of m6A regulators in eutopic endometrium of infertile EMS patients and identification of key diagnostic candidate regulators

**Figure 1.**
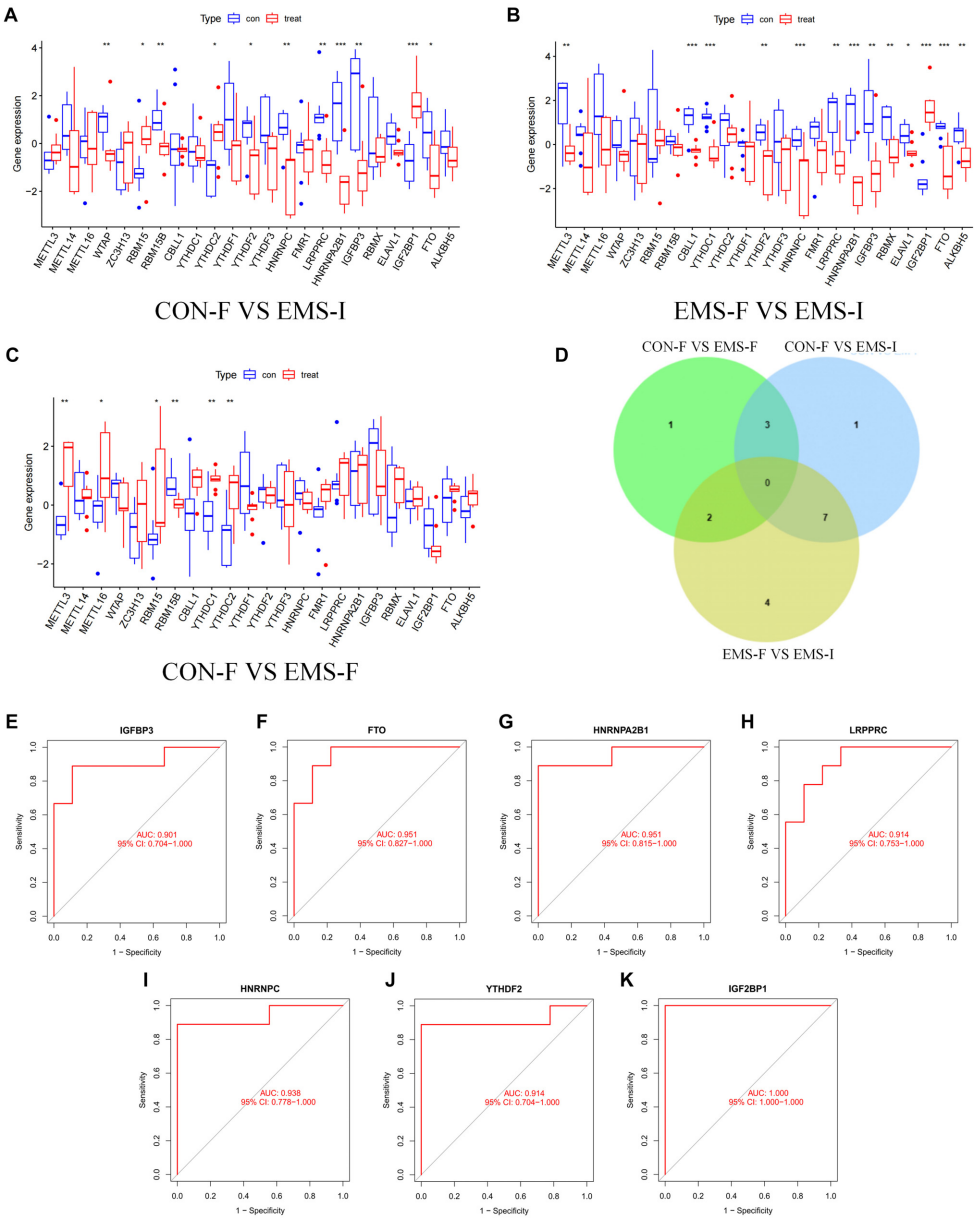
**Expression features of m6A regulators in eutopic endometrium from infertile patients with EMS and identification of key diagnostic candidate regulators**. (A) Differences in the expression of m6A regulators in endometrial tissue between normal fertile women and infertile patients with EMS; (B) Differences in the expression of m6A regulators in endometrial tissue between fertile and infertile patients with EMS; (C) Differences in the expression of m6A regulators in endometrial tissue between normal fertile women and fertile EMS patients; (D) Correlation analysis of differentially expressed m6A regulators in the endometrial tissue of normal fertile women, fertile EMS patients, and infertile EMS patients; (E–K) ROC curve analysis of intersecting m6A regulators. m6a: N6-methyladenosine; EMS: Endometriosis; ROC: Receiver operating characteristic.

This study analyzed eutopic endometrium samples obtained from nine normal fertile women and 18 stage IV ovarian EMS patients (nine fertile and nine infertile) using data from the GSE120103 dataset. The expression levels of m6A regulators were examined, and box plots were generated for visualization [15]. As shown in Figure 1A–1C, 11, 13, and 6 m6A regulators with significant expression differences were identified when comparing (1) normal fertile women and infertile EMS patients, (2) fertile EMS and infertile EMS patients, and (3) normal fertile women and fertile EMS patients, respectively.

To identify specific m6A regulators associated with infertility in EMS patients while excluding the potential confounding effects of EMS on fertility, an intersection analysis of these three sets of DEGs was performed. This analysis identified seven m6A regulators: *IGFBP3*, *FTO*, *HNRNPA2B1*, *LRPPRC*, *HNRNPC*, *YTHDF2*, and *IGF2BP1* (Figure 1D).

Additionally, receiver operating characteristic (ROC) curve analysis was conducted for these seven regulators to evaluate their predictive ability and diagnostic value based on AUC values (Figure 1E–1K). The results showed that all seven regulators had AUC values greater than 0.9, demonstrating significant diagnostic value for EMS-related infertility. These regulators were subsequently included in related analyses.

### Construction of the nomogram model for key candidate m6A regulators and identification of molecular clusters via unsupervised clustering

**Figure 2.**
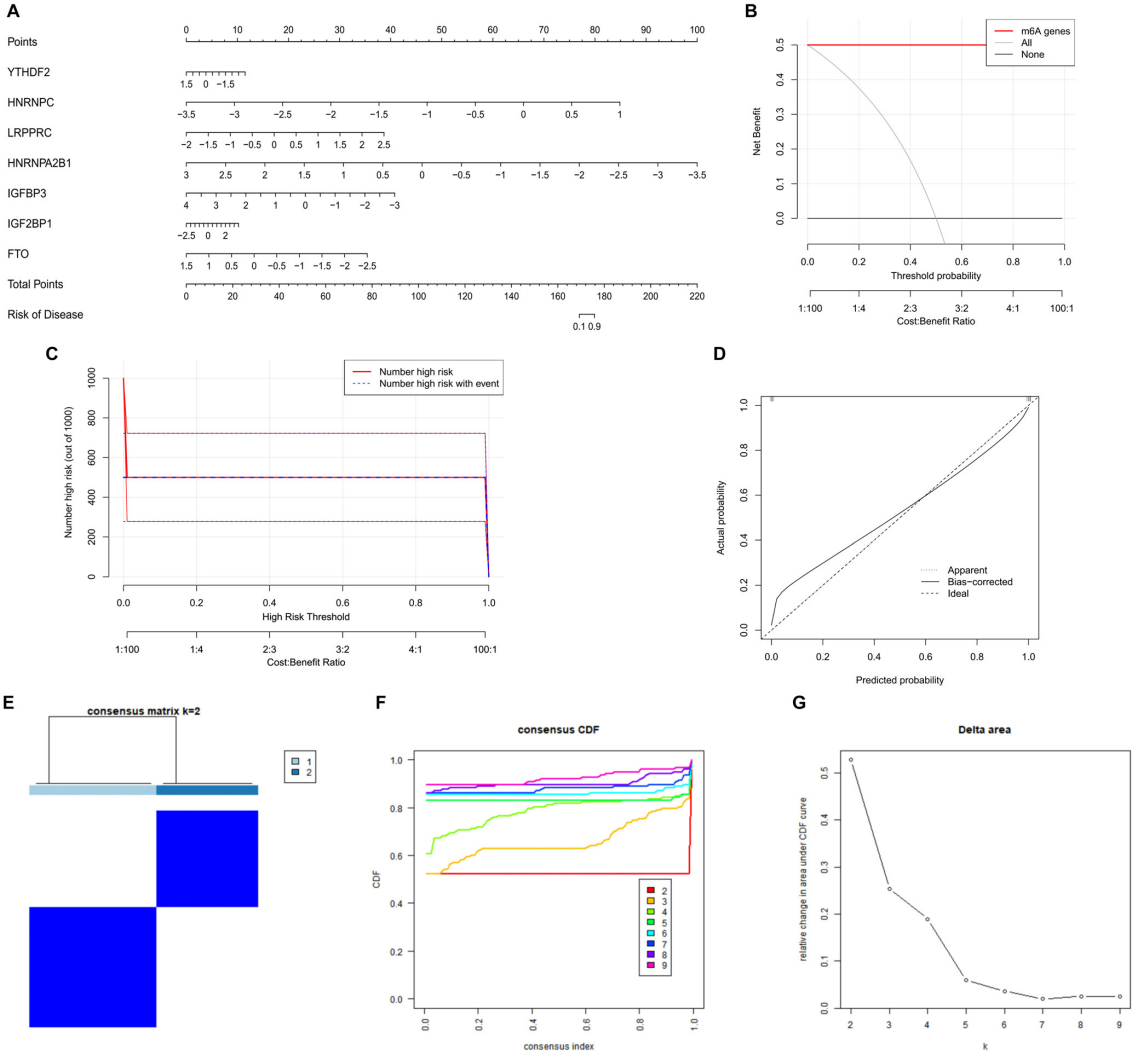
**Construction of the nomogram model for key candidate m6A regulators and identification of molecular clusters via unsupervised clustering.** (A) Nomogram model based on key candidate m6A regulators; (B) DCA curve of the nomogram model; (C) The clinical impact curve of the nomogram model; (D) Calibration curve of the nomogram model; (E) m6A molecular clusters based on candidate regulators with K ═ 2; (F) The CDF curve (K ═ 2–9); (G) The variation in the area under CDF curve (K ═ 2–9). m6a: N6-methyladenosine; DCA: Decision curve analysis.

A nomogram model based on key candidate regulators was constructed using the “rms” package in R software, with *HNRNPA2B1* having the most significant effect (Figure 2A). In the DCA curve, the red and black lines are clearly distinct, demonstrating that decisions informed by the nomogram model are valuable for assessing the reproductive capacity of EMS patients (Figure 2B). The clinical impact curve further supports the model’s predictive ability, with the prediction curve closely aligning with the actual outcomes (Figure 2C). Additionally, the calibration curve shows strong agreement among the predicted dashed line, the actual solid line, and the bootstrap (1000 repetitions) thick solid line, confirming that the nomogram model is reliable, accurate, and predictive (Figure 2D).

**Figure 3.**
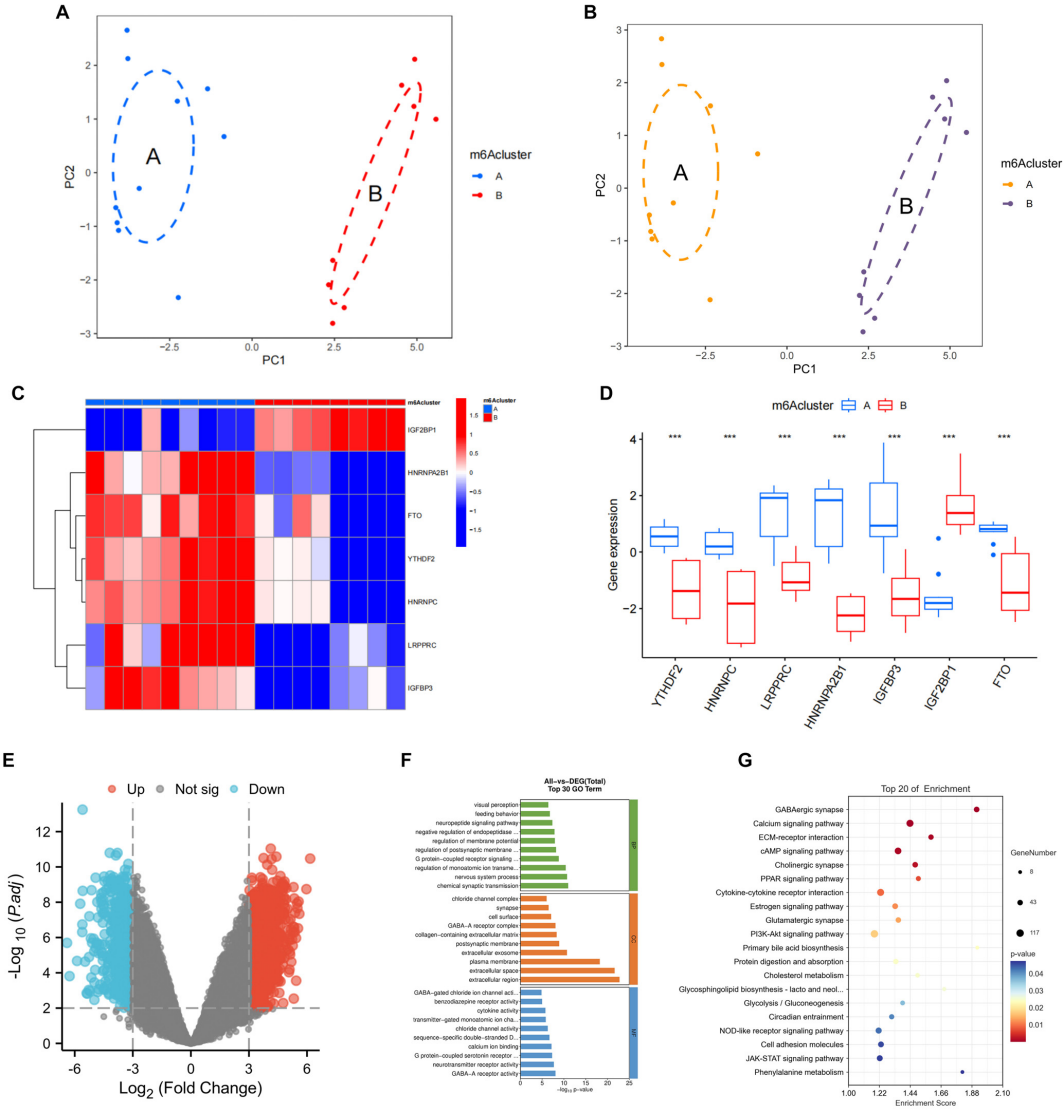
**Cluster grouping of key candidate m6A regulators and GO and KEGG analyses of DEGs.** (A) PCA of two m6A clusters; (B) PCA of the adjusted two clusters; (C) Clustering heatmap of key candidate m6A regulators in the adjusted two clusters; (D) Differences in the expression levels of key candidate m6A regulators in the adjusted two clusters; (E) Statistical plot of DEGs in the adjusted two clusters; (F) GO analysis of DEGs in the adjusted two clusters (top 30 GO terms); (G) KEGG analysis of DEGs in the adjusted two clusters (top 20 pathways). m6a: N6-methyladenosine; GO: Gene Ontology; DEG: Differentially expressed gene; KEGG: Kyoto Encyclopedia of Genes and Genomes; PCA: Principal component analysis.

Using the “ConsensusClusterPlus” package and unsupervised clustering with K values ranging from 2 to 9, distinct m6A molecular clusters among the candidate regulators were identified (Figure 2E). The area under the CDF curve suggests that stable sample separation begins at three clusters (Figure 2F and 2G), while PCA indicates near-perfect separation into two clusters (Figure 3A). Based on these findings, *K* ═ 2 was selected for the m6A classification of EMS samples. As shown in Table 1, Cluster A consists of 10 samples, and Cluster B consists of eight samples. Notably, the cluster grouping largely corresponds to the fertile and infertile classifications of the donors, with only one sample misclassified.

**Table 1:** Relationship between the m6A cluster and reproductive capacity

**ID**	**Reproductive capacity**	**m6A cluster**
GSM3393500	Fertile	A
GSM3393501	Fertile	A
GSM3393502	Fertile	A
GSM3393503	Fertile	A
GSM3393504	Fertile	A
GSM3393505	Fertile	A
GSM3393506	Fertile	A
GSM3393507	Fertile	A
GSM3393508	Fertile	A
GSM3393518	Infertile	B
GSM3393519	Infertile	B
GSM3393520	Infertile	B
GSM3393521	Infertile	B
GSM3393522	Infertile	B
GSM3393523	Infertile	B
GSM3393524	Infertile	B
GSM3393525	Infertile	A
GSM3393526	Infertile	B

Based on the candidate key m6A regulators (*IGFBP3*, *FTO*, *HNRNPA2B1*, *LRPPRC*, *HNRNPC*, *YTHDF2*, and *IGF2BP1*), the samples of 18 stage IV ovarian EMS patients (9 fertile and 9 infertile) in GSE120103 database were classified into 2 m6A clusters (Cluster A and Cluster B) by utilizing “ConsensusClusterPlus” package in R, with Cluster A containing 10 samples and Cluster B containing 8 samples. The cluster grouping is largely consistent with the fertile and infertile classification of the sample donors, with only 1 sample misclassified (sample ID: GSM3393525). m6a: N6-methyladenosine.

### Cluster grouping of key candidate m6A regulators and GO and KEGG analyses of DEGs

After *K* ═ 2 was applied for m6A classification of the EMS samples, one infertile sample was found to be inconsistent with its assigned cluster. As a result, this study excluded that sample and retained only the correctly classified samples for further analysis. The PCA results for the adjusted two clusters are displayed in Figure 3B. The clustering heatmap (Figure 3C) shows that the key candidate m6A regulators cluster cohesively within the adjusted clusters, with statistically significant differences in the expression levels of these m6A regulators between the groups (*P* < 0.001) (Figure 3D). Further statistical analysis of the DEGs in the adjusted clusters (Figure 3E) revealed 2354 DEGs with a *P* value of 0.001 and logFC ═ 3. GO and KEGG analyses were subsequently performed on the identified DEGs, with results presented in Figure 3F and 3G. GO analysis indicated that the DEGs are primarily involved in immune signal transduction, signaling molecules, and interaction processes. In contrast, KEGG analysis revealed enrichment of DEGs in pathways related to endocrine signaling, immune cell adhesion, and glucose and lipid metabolism. The top 20 pathways include the oestrogen signaling pathway, cAMP signaling pathway, cell adhesion molecules, and glycolysis/gluconeogenesis, among others.

### Relationships among key candidate m6A regulators, their cluster grouping and immune cell infiltration

**Figure 4.**
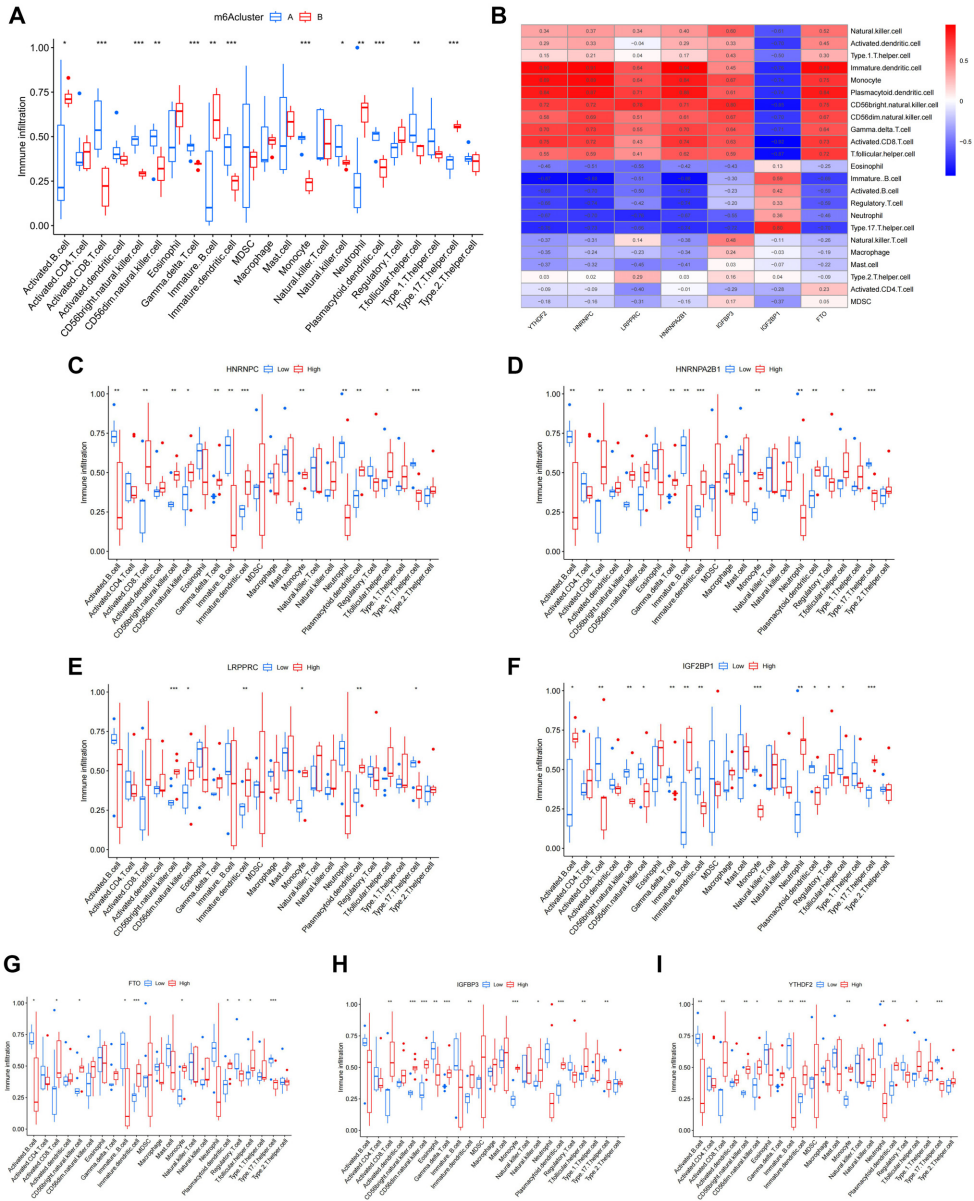
**Relationships among key candidate m6A regulators, cluster grouping and immune cell infiltration.** (A) The correlation between the two m6A clusters and immune cell infiltration; (B) Correlations between key candidate m6A regulators and immune cell infiltration; (C) Differences in immune cell infiltration between groups with low and high *HNRNPC* expression; (D) Differences in immune cell infiltration between groups with low and high *HNRNPA2B1* expression; (E) Differences in immune cell infiltration between groups with low and high *LRPPRC* expression; (F) Differences in immune cell infiltration between groups with low and high *IGF2BP1* expression; (G) Differences in immune cell infiltration between groups with low and high *FTO* expression; (H) Differences in immune cell infiltration between groups with low and high *IGFBP3* expression; (I) Differences in immune cell infiltration between groups with low and high *YTHDF2* expression. m6a: N6-methyladenosine.

Through GO and KEGG analyses, this study revealed that the clustering of m6A regulators is closely associated with immune pathways. Consequently, an analysis of immune cell infiltration in m6A clusters was performed (Figure 4A). The results showed that immune cell infiltration levels in Cluster A were significantly lower than those in Cluster B, with the most pronounced differences observed in activated B cells, immature B cells, neutrophils, and type 17 T helper cells. Additionally, ssGSEA was employed to assess the correlation between key candidate m6A regulators and immune cell infiltration (Figure 4B). Several regulators demonstrated significant positive correlations with CD56 bright natural killer (NK) cells, monocytes, plasmacytoid dendritic cells, immature dendritic cells, and activated CD8+ T cells, with *HNRNPC*, *HNRNPA2B1*, *YTHDF2*, and *FTO* standing out prominently. Lastly, the study examined differences in immune cell infiltration in the eutopic endometrium between donors with low and high expression levels of m6A regulators (Figure 4C–4I). The findings revealed that m6A regulator expression significantly influences immune cell infiltration, with marked effects observed in *HNRNPC*, *HNRNPA2B1*, *IGF2BP1*, *IGFBP3*, and *YTHDF2*.

**Figure 5.**
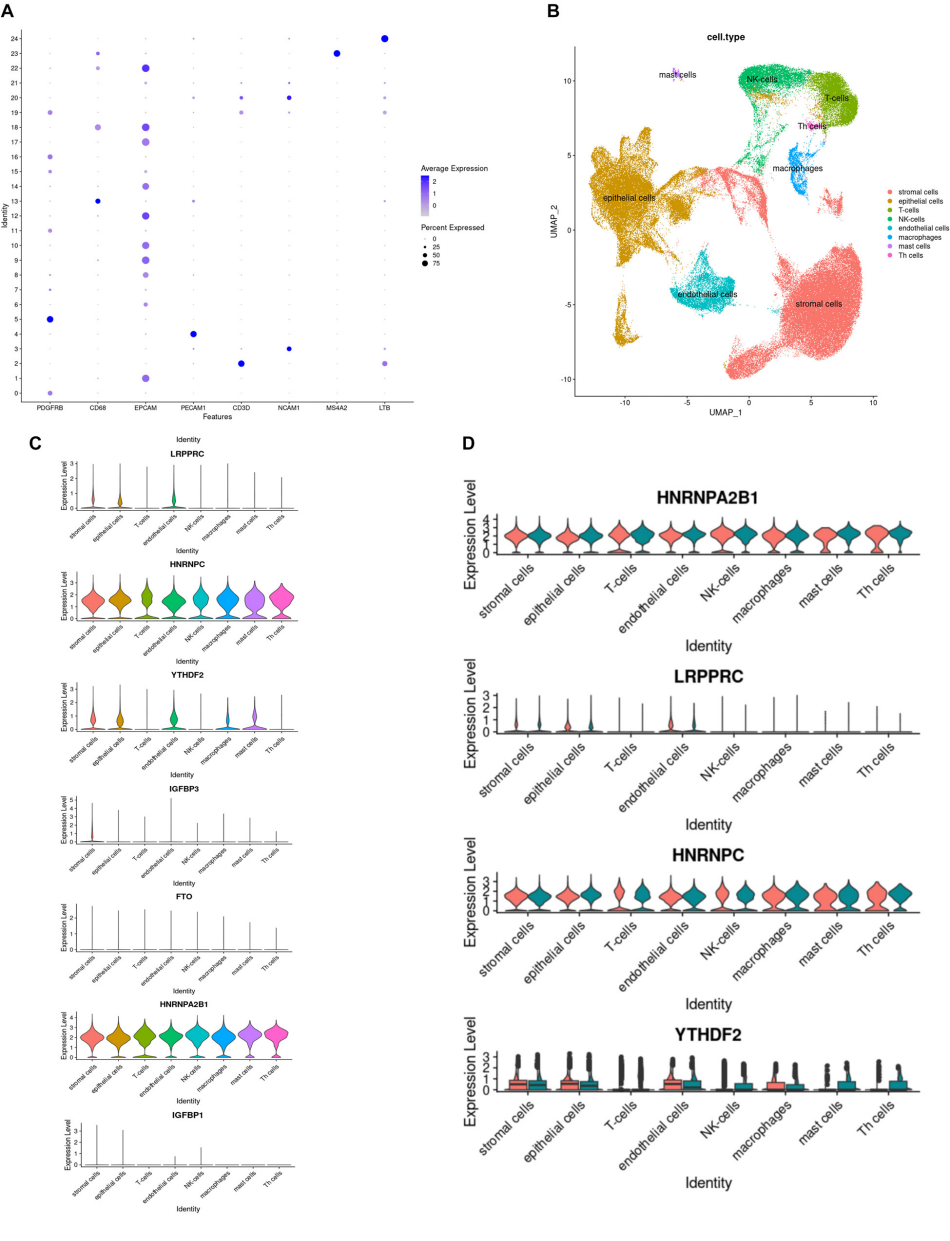
**Analysis of the expression levels of key candidate m6A regulators in various cell types.** (A) The characteristically expressed genes of different cell types; (B) Distribution of the expression levels of various cell types; (C) The expression levels of each key candidate m6A regulator in various cell types of eutopic endometrium; (D) The expression levels of *HNRNPA2B1*, *HNRNPC*, *YTHDF2*, and *LRPPRC* in various cell types within eutopic endometrium of normal fertile women and infertile EMS patients. m6a: N6-methyladenosine.

**Figure 6.**
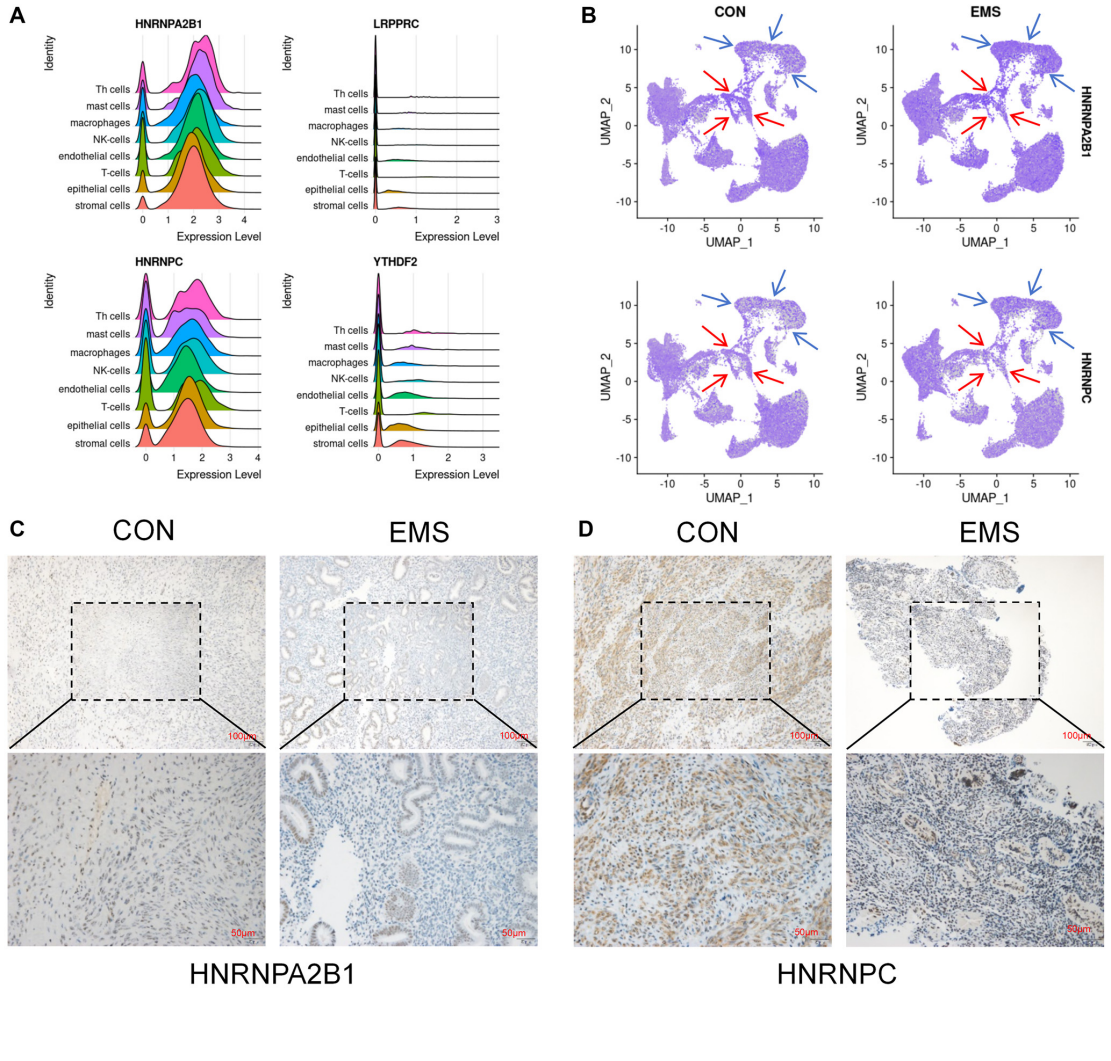
***HNRNPA2B1* and *HNRNPC* can serve as potential biomarkers for EMS-related infertility.** (A) Sierra figures of the expression levels of *HNRNPA2B1*, *HNRNPC*, *YTHDF2*, and *LRPPRC* in various cell types within eutopic endometrium of normal fertile women and infertile patients with EMS; (B) Analysis of the expression levels of *HNRNPA2B1* and *HNRNPC* in various cell types in the eutopic endometrium of normal fertile women and infertile patients with EMS. The immune cells are marked by blue arrows, and the stromal cells are marked by red arrows; (C) Immunohistochemical staining analysis of HNRNPA2B1 in eutopic endometrium from normal fertile women and infertile patients with EMS; (D) Immunohistochemical staining analysis of HNRNPC in eutopic endometrium from normal fertile women and infertile patients with EMS. EMS: Endometriosis; NK: Natural killer.

### Analysis of the expression levels of key candidate m6A regulators in various cell types

To further investigate the role of key candidate m6A regulators in the functions of various cell types, this study categorized a single-cell dataset (GSE214411) based on the characteristic expression of genes specific to different cell types (Figure 5A). Cell type-specific markers were identified by referencing research conducted by Huang et al. [14]. The classification results for the cell types are presented in Figure 5B. Subsequently, the expression levels of each key candidate m6A regulator in various cell types of the eutopic endometrium were analyzed (Figure 5C). The analysis revealed that three key candidate m6A regulators—*IGF2BP1*, *IGFBP3*, and *FTO*—were almost undetectable across all cell types. This low detection could be attributed to the quality control parameters applied to the single-cell data. Therefore, the study focused on analyzing the expression levels of the remaining four key candidate m6A regulators in various cell types of the eutopic endometrium for both the normal fertility and EMS infertility groups (Figure 5D). The results showed that the expression levels of *HNRNPA2B1* and *HNRNPC* were significantly higher than those of *YTHDF2* and *LRPPRC* across all analyzed cell types.

### *HNRNPA2B1* and *HNRNPC* can serve as potential biomarkers for EMS-related infertility

This study used Sierra figures to analyze the expression levels of the remaining four key candidate m6A regulators in various cell types within the eutopic endometrium of normal fertile women and infertile patients with EMS (Figure 6A). The results revealed that *HNRNPA2B1* and *HNRNPC* were expressed at high levels in all cell types within the eutopic endometrium of normal fertile women and infertile patients with EMS, with significant intergroup differences, whereas *YTHDF2* and *LRPPRC* presented lower expression levels with no significant intergroup differences. Therefore, *YTHDF2* and *LRPPRC* were excluded from the analysis. The expression levels of *HNRNPA2B1* and *HNRNPC* in various cell types within the eutopic endometrium of normal fertile women and infertile patients with EMS were analyzed (Figure 6B). The results revealed that *HNRNPA2B1* and *HNRNPC* were significantly elevated in immune cells from the endometrial tissue of infertile patients with EMS but significantly decreased in stromal cells. Finally, clinical samples of eutopic endometrium from normal fertile women and infertile ovarian patients with EMS were collected, and immunohistochemical staining was used to analyze the expression levels of HNRNPA2B1 and HNRNPC (Figure 6C and 6D). The results indicated that the expression levels of HNRNPA2B1 and HNRNPC in endometrial tissue from normal fertile women were significantly higher than those in ovarian tissue from infertile women with EMS. In summary, this study predicted that *HNRNPA2B1* and *HNRNPC* could serve as potential biomarkers of EMS-related infertility.

## Discussion

EMS is an inflammatory, estrogen-dependent disease strongly associated with pelvic pain and infertility [16]. Among its clinical manifestations, EMS-related infertility has attracted significant attention, particularly due to its impact on reproductive capacity, which is the focus of the present study [17–19]. Recently, RNA modifications, particularly m6A methylation, have emerged as key topics in research on female reproductive diseases, including EMS, infertility, premature ovarian failure, PCOS, and adenomyosis [20]. Despite these advancements, the precise role of m6A methylation in EMS and its contribution to infertility remains unclear. To address this gap, the present study investigated m6A methylation, employing bioinformatics and bulk-sequencing technology to analyze the expression levels of m6A regulators in the eutopic endometrium of EMS patients. Additionally, key biomarkers were identified to guide the diagnosis and clinical management of EMS-related infertility.

Using the GSE120103 dataset, this study identified specific m6A regulators associated with EMS-related infertility and demonstrated their diagnostic value through ROC curve analysis. Subsequently, a nomogram model was constructed, and unsupervised clustering analysis identified distinct m6A clusters. The findings suggested that m6A methylation plays a crucial role in EMS-related infertility. Functional enrichment analysis of DEGs between the two m6A clusters revealed significant enrichment in immune cell-related pathways. Further immune cell infiltration analysis showed that m6A regulators—particularly *HNRNPC*, *HNRNPA2B1*, *IGF2BP1*, *IGFBP3*, and *YTHDF2*—profoundly influence immune cell infiltration. Notably, immune imbalance under EMS conditions is strongly associated with infertility. Maternal immune tolerance, largely mediated by regulatory T cells (Tregs), is essential for successful pregnancy. Tregs suppress effector immune responses, regulate inflammation, and support maternal vascular adaptation, enabling trophoblast invasion and placental access to the maternal blood supply. However, insufficient numbers or functional impairment of Tregs can result in idiopathic infertility and recurrent miscarriage [21]. In EMS patients, certain endometrial immune cells exhibit cyclic phase changes similar to those in healthy women; however, significant differences are observed in macrophages (Mø), immature dendritic cells, and Tregs. Pro-inflammatory Mø1s and anti-inflammatory Mø2s dominate at different stages, while NK cells in the endometrium of EMS patients display abnormal activity levels. These changes contribute to an aberrant inflammatory state in the endometrium, ultimately leading to infertility [22]. Based on these findings, this study speculates that the expression of key m6A regulatory factors in the eutopic endometrium significantly influences immune cell infiltration. This, in turn, disrupts immune balance and promotes an abnormal inflammatory state, culminating in EMS-related infertility.

In addition to immune cell-related pathways, endocrine-related pathways were significantly enriched. Among these, the ovarian estrogen signaling pathway was notably prominent. As an estrogen-dependent disease, endometriotic ectopic lesions in EMS contain key enzymes involved in estrogen synthesis, and excessive estrogen promotes ectopic lesion growth. In EMS patients, estrogen dominance disrupts the interaction between progesterone and estrogen signaling, often leading to progesterone resistance. This hormonal imbalance exacerbates inflammation, increases pelvic pain, and reduces endometrial receptivity, ultimately contributing to infertility [23].

Ovarian EMS, characterized by endometriotic lesions on the ovary, has been shown to negatively impact ovarian physiology. Ultrasound and histological data reveal a reduced number of ovarian follicles and increased follicular atresia in EMS patients. Additionally, the local follicular environment in these patients shows granulosa cell alterations, including reduced P450 aromatase expression and elevated intracellular reactive oxygen species (ROS), which further impair follicle maturation [24]. Our research team previously reported that cyclic bleeding of ovarian ectopic lesions creates a localized iron-overloaded environment, leading to ferroptosis in granulosa cells and oocyte immaturity. This contributes to EMS-related infertility [25]. The expression levels of key m6A regulators in the eutopic endometrium may significantly impact the estrogen-progesterone balance, exacerbating inflammation, accelerating ectopic lesion growth, and reducing both endometrial receptivity and ovarian function.

To further investigate the role of key m6A regulators in various cell types, this study analyzed the single-cell dataset GSE214411. The results showed that *HNRNPA2B1* and *HNRNPC* expression levels were significantly higher in endometrial immune cells from infertile EMS patients but lower in stromal cells. Prior analysis also indicated that key m6A regulator expression was higher in Cluster A (fertile EMS group) compared to Cluster B (infertile EMS group), mirroring the expression patterns of *HNRNPA2B1* and *HNRNPC* in stromal cells of the endometrium in fertile women and ovarian EMS patients. This suggests that reduced expression of *HNRNPA2B1* and *HNRNPC* in stromal cells—key components of endometrial tissue—may serve as diagnostic markers for EMS-related infertility.

Clinical samples analyzed in this study confirmed these findings via immunohistochemistry, revealing significantly higher expression of HNRNPA2B1 and HNRNPC in the eutopic endometrium of fertile women compared to ovarian EMS patients. These findings suggest that *HNRNPA2B1* and *HNRNPC* play vital roles in female reproductive ability. However, the mechanisms by which these regulators function remain unclear and warrant further exploration. In summary, this study proposes *HNRNPA2B1* and *HNRNPC* as potential biomarkers for EMS-related infertility. By leveraging multiple public datasets, the findings suggest clinical significance for diagnosis. However, several limitations remain. The small sample size, constrained by public database availability, underscores the need for additional clinical samples to validate these conclusions. Furthermore, current methylation detection in EMS relies on surgically obtained samples. Future research should prioritize non-invasive approaches, such as analyzing menstrual effluent or blood biomarkers, for early EMS diagnosis [26, 27]. Lastly, this study relied primarily on data analysis. Complementary biological research, including *in vitro* and *in vivo* experiments, is needed to elucidate the molecular mechanisms of m6A regulators and assess their clinical potential.

Notably, this is the first study to categorize EMS based on reproductive capacity using methylation analysis—an unexplored avenue in EMS research. Since EMS-related infertility diagnoses are often delayed, detecting key m6A regulators in endometrial samples obtained through simple uterine curettage could offer a novel and early diagnostic approach. This would enable timely fertility counseling for patients.

## Conclusion

M6A regulators appear to play significant roles in the occurrence and progression of EMS-related infertility. Additionally, *HNRNPA2B1* and *HNRNPC* have potential as biomarkers for diagnosing or monitoring EMS-related infertility.

## Data Availability

The GSE120103 RNA sequencing dataset was obtained from https://www.ncbi.nlm.nih.gov/geo/query/acc.cgi?acc=GSE120103. The GSE214411 single-cell dataset was obtained from https://www.ncbi.nlm.nih.gov/geo/query/acc.cgi?acc=GSE214411. The remaining experimental data can be available from the corresponding authors on reasonable request.
